# Chronic post-surgical pain in older adults: mechanisms, assessment, and management strategies

**DOI:** 10.3389/fmed.2025.1719477

**Published:** 2026-01-12

**Authors:** Jiyu Zeng, Ting Yang, Jiaman Li

**Affiliations:** 1 Department of Anesthesiology, West China Hospital of Sichuan University Ziyang Hospital, Ziyang, Sichuan, China; 2 Department of Medical Education, West China Hospital of Sichuan University Ziyang Hospital, Ziyang, Sichuan, China

**Keywords:** chronic postoperative pain, older adults, pain mechanism, prevention, treatment

## Abstract

Chronic postsurgical pain (CPSP) in older adults is a multifactorial condition shaped by biological, psychological, and social determinants. This review synthesizes current evidence regarding the underlying mechanisms, clinical assessment, and management strategies for CPSP in the older adults. Key findings emphasize age related changes in pain processing, significant challenges in pain evaluation particularly among those with cognitive impairment, and the limited effectiveness of conventional analgesic therapies. Multimodal and personalized strategies, such as regional anesthesia, tailored pharmacologic interventions, and integrative approaches including acupuncture, demonstrate potential in reducing the incidence and burden of CPSP. Implementing structured assessment protocols and comprehensive geriatric care models is crucial for enhancing postoperative quality of life. Future initiatives should focus on integrating predictive tools, long term monitoring systems, and interdisciplinary collaboration to improve pain related outcomes in this vulnerable population.

## 1 Introduction

Chronic postsurgical pain (CPSP), defined as pain lasting over 3 months after surgery unrelated to prior conditions (1), is a growing concern, especially among the rapidly increasing older population (2, 3). This population is experiencing a rising incidence of surgical interventions and demonstrates a heightened vulnerability to postoperative complications, including chronic pain, which poses distinct management challenges for older adults (4–6). The physiological and metabolic characteristics inherent to older patients result in pain perception and response that differ significantly from those of younger individuals, thereby exacerbating the complexities of pain management. Furthermore, the management of pain in older patients is further complicated by the frequent presence of cognitive disorders and multiple chronic conditions (such as osteoarthritis, diabetic neuropathy, cardiovascular diseases, and frailty syndrome), which often coexist with pain (7).

The management of chronic pain in older patients requires a comprehensive approach that integrates biological, psychological, and social dimensions. An optimal strategy for pain management involves a multi-modal treatment plan that emphasizes interdisciplinary collaboration to improve patients’ quality of life and autonomy (8). It is particularly important to identify and assess pain in patients with cognitive impairments, as these individuals may face challenges in effectively communicating their pain (7).

Although a variety of interventions exist for managing CPSP, traditional therapeutic approaches frequently demonstrate limited efficacy and can lead to significant side effects, particularly in the older adults. This challenge is compounded in older adults by complexities of pharmacotherapy administration, including drug interactions, polypharmacy, and altered drug metabolism, often resulting in suboptimal pain control and heightened risk of adverse reactions (9). Given the rising frequency of surgeries among the older adults and the substantial burden of CPSP, a thorough understanding of this condition is crucial. However, the limitations of current strategies highlight a critical need to investigate safer and more effective alternatives, specifically emphasizing personalized, multi-modal pain management programs for this vulnerable population. Consequently, this paper aims to provide a review and synthesis of recent research on CPSP in the older adults, focusing on its epidemiology, underlying mechanisms, assessment methods, treatment, and prevention. By critically analyzing current research challenges and identifying gaps, this study seeks to inform future academic investigations and clinical practices. The ultimate goal is to advance the development and implementation of effective strategies, thereby improving postoperative quality of life for older patients and reducing the strain on healthcare resources.

## 2 Epidemiology of CPSP in the older adults

### 2.1 The growing trend of the older population and its impact on the healthcare system

As the global trend of population aging intensifies, the number of older individuals is increasing rapidly, and the health challenges they encounter are becoming more pronounced. According to United Nations projections, the global population of individuals aged 60 and over is expected to reach 2.1 billion by 2050, representing a significant demographic shift. This trend presents an unprecedented challenge to the global healthcare system. The demographic shift toward an aging population is anticipated to significantly increase healthcare utilization and associated costs. Age-related conditions, particularly chronic diseases, are key drivers of this heightened demand for health services. A survey conducted by Lily et al. specifically examining chronic pain demonstrated a significant correlation with increased hospitalization frequency (IRR 1.10, 95% CI: 1.01, 1.31), reflecting its substantial impact on acute medical resource utilization (10). This finding aligns with broader evidence from developed nations, where chronic pain consistently contributes to elevated health service usage patterns (10).

### 2.2 Prevalence and epidemiological characteristics of CPSP in the older adults

Chronic postsurgical pain substantially affects the quality of postoperative recovery in older patients. Research conducted a 15 years ago estimated that over 19 million (11) (19 million = 37.36% of 51 million surgeries) older individuals undergo surgical procedures annually in the United States, with 10%–60% experiencing CPSP (12–14). A study by Gary et al. similarly reported a 40% (OR 4.73, 95% CI 1.24, 18.09) incidence of CPSP among older frail patients after surgery (15). The research elevated incidence rate can be partially attributed to age-related degeneration of the nervous system, alterations in pain perception, and a diminished capacity for postoperative recovery in older adults (16). Furthermore, older patients frequently suffer from chronic conditions such as arthritis and diabetes, which can influence the transmission and perception of pain signals through intricate mechanisms, thereby elevating the risk of developing CPSP. Epidemiological research has indicated that older women are more susceptible to CPSP compared to their male counterparts, a disparity potentially attributable to physiological and metabolic differences, as well as lifestyle factors (3). Evidence suggests that female gender may be an independent risk factor for chronic postoperative pain following thoracoscopic surgery (17). This association is mediated through multiple mechanisms. Biologically, sex-based variations exist in hormone levels, the activity of key nociceptive receptors (including N-methyl-D-aspartate and P2×3 receptors), the distribution of μ/κ opioid receptor subtypes within endogenous pain modulation pathways, and sexually dimorphic neuroanatomy and neural processing (18). Psychologically, females exhibit heightened pain awareness and expressiveness, resulting in increased pain reporting (19). Additionally, it is important to acknowledge that regional cultural variations and disparities in medical resources also contribute to variations in CPSP statistics (20).

## 3 Age-related mechanistic vulnerabilities in pain processing

### 3.1 Biological aging and pain sensitivity

Chronic postsurgical pain is more prevalent among older patients, attributed to a range of biological factors. Research suggests that age-related physiological changes, such as nervous system degeneration, immune function decline, and the presence of chronic diseases, may heighten the risk of developing postoperative chronic pain (21). Furthermore, older patients frequently present with multiple comorbidities such as osteoarthritis, diabetes mellitus with neuropathic complications, and chronic kidney disease, which can influence pain perception and processing, thereby exacerbating postoperative pain (22). Pain catastrophizing refers to a cognitive distortion characterized by the amplification of and persistent rumination on pain sensations (23). This tendency leads to an exaggerated perception of threat-related components within the pain experience, thereby influencing self-reported pain intensity and pain-associated psychological states (24). Consequently, the interaction between pain catastrophizing and negative emotions may exacerbate distress, anger, fear, frustration, or anxiety. These emotional responses play a critical role in the context of chronic pain and the overall management of pain (24).

Biological factors encompass genetic predispositions and alterations in biomarkers. Specific gene variants have been associated with pain sensitivity and the development of chronic pain (25).

The transition from acute to chronic pain involves highly complex pathological mechanisms, prominently featuring peripheral and central sensitization phenomena (26). Peripheral sensitization denotes an enhanced responsiveness and reduced activation threshold of nociceptive neurons within peripheral tissues to stimulation of their receptive fields (27). Central sensitization, conversely, refers to an increased responsiveness of nociceptive neurons within the central nervous system to both normal and subthreshold afferent input (27).

Surgical tissue injury plays a fundamental role in the development of CPSP, underpinning significant neuroplastic alterations within peripheral and central sensory neural circuits. Nociceptive input into the spinal cord dorsal horn (SCDH) triggers the release of the neurotransmitter glutamate. Glutamate subsequently acts on specific postsynaptic receptors, including α-amino-3-hydroxy-5-methyl-4-isoxazolepropionic acid (AMPA) receptors and the critically involved N-methyl-D-aspartate receptors (NMDARs). Sustained and intense glutamate release in the SCDH, driven by persistent peripheral afferent barrage following surgical nerve injury, leads to NMDAR activation. This receptor activation facilitates synaptic plasticity and, under conditions of severe or prolonged stimulation, can contribute to neurotoxicity and neuronal apoptosis (28).

As a direct consequence of these sensitization processes, patients developing CPSP frequently exhibit characteristic clinical manifestations early in the postoperative period, including hyperalgesia (heightened pain response to noxious stimuli), allodynia (pain elicited by normally innocuous stimuli), and dysesthesias (unpleasant abnormal sensations) (26).

Aging is associated with a decline in the organism’s capacity to accurately identify noxious signals, while pain tolerance remains static or may even diminish. Paradoxically, upon detection of a noxious stimulus, an exaggerated pain response often occurs. Consequently, older individuals experiencing acute pain are highly susceptible to its rapid progression to severe, often intractable, pain states (29).

The mechanisms of peripheral and central sensitization, common in chronic pain, may develop more subtly in older adults due to reduced neuroplasticity with age. Most evidence comes from mixed-age groups or preclinical studies, with few direct neurobiological studies on older CPSP patients, highlighting a key area for future research.

### 3.2 Inflammaging and immune dysregulation

The mechanisms of inflammation and immune regulation in chronic pain are especially critical in geriatric patients due to the substantial alterations in immune function associated with aging. Post-operative inflammation has the potential to result in persistent pain. Increasing evidence indicates that inflammatory mediators associated with the nuclear factor κB pathway are involved in pain processes within central and peripheral nervous systems, including neurons and glial cells (particularly astrocytes, which are a major type of central glial cell) (30, 31). Interleukin-6, a cytokine associated with adverse outcomes in older adults, is implicated in both pain perception and the development of pathological pain (32, 33). However, research has demonstrated a significant correlation between increased circulating levels of pro-inflammatory cytokines (IL-8, IL-10, IL-18) and chemokines [e.g., C-C motif chemokine ligand 2 (CCL2), C-X-C motif chemokine ligand 10 (CXCL10)] and the severity of pain across various pain syndromes. CCL2, also known as monocyte chemoattractant protein 1 (MCP-1), recruits monocytes and microglia to the site of nerve injury or inflammation, activating neuroinflammatory cascades that promote central sensitization. CXCL10 enhances nociceptive signaling by binding to CXCR3 receptors on neurons, increasing neuronal excitability (34–36). The role of nuclear factor κB as a mediator in the regulation of post-surgical pain remains to be elucidated. Clinical studies suggest that plasma concentrations of inflammatory mediators generally change following surgery and may increase immediately thereafter (37–39). Substance P in neurogenic inflammation has been extensively validated in multiple studies. By binding to its receptor, neuropeptide-1 receptor (NK1R), substance P promotes the activation of inflammatory cells and the release of chemokines, thereby exacerbating inflammatory responses (40). In facial pain studies, CGRP (calcitonin gene-related peptide) amplifies pain by upregulating nitric oxide synthase expression, a process involving the p38 signaling pathway. This mechanism may also apply to postoperative pain, as it is frequently accompanied by inflammatory responses, and CGRP has been shown to contribute to inflammation-mediated pain (41). Postoperative inflammatory responses can play a critical role in the development of CPSP. In older individuals, the inflammatory response is frequently characterized by a low-grade chronic inflammatory state, which may result in an amplified postoperative inflammatory response. This heightened response can adversely affect wound healing and contribute to the induction of chronic pain.

Furthermore, immune dysregulation in the older adults often disrupts the equilibrium between pro-inflammatory and anti-inflammatory responses, potentially exacerbating the experience of chronic pain (42). Regulatory T cells (Treg cells) play a vital role in maintaining immune tolerance and modulating inflammatory responses. Following spinal cord injury, Treg cells attenuate neuropathic pain by suppressing neuroinflammation (43). This immunomodulation occurs primarily through Treg-mediated release of inhibitory cytokines and functional regulation of other immune cells. However, experimental depletion of Treg cells exacerbates mechanical allodynia (hypersensitivity to innocuous mechanical stimuli) and significantly alters systemic cytokine profiles (44). Emerging research suggests that specific probiotics have the potential to mitigate chronic pain symptoms by modulating the immune response, thereby presenting a novel strategy for managing postoperative pain in the older population (45). Aging induces modifications in immune system functionality, marked by changes in cytokine concentrations and immune cell activity. These immune alterations may affect the neurological milieu at the postoperative wound site, potentially facilitating chronic pain through complex neuro-immune interactions.

Mitochondrial dysfunction and oxidative stress are increasingly implicated in CPSP pathogenesis. Evidence indicates that mitochondrial impairment drives reactive oxygen species (ROS) accumulation, subsequently inducing neuronal pyroptosis – an inflammatory programmed cell death process that critically contributes to CPSP (46). Specifically, downregulation of mitofusin-2 (Mfn2) associates with mitochondrial dysfunction and ROS overproduction. These pathological changes promote pyroptosis in spinal GABAergic neurons, thereby facilitating chronic pain development (46).

Age-related reductions in peroxisome proliferator-activated receptor gamma coactivator 1-alpha (PGC-1α) expression further contribute to pain chronification. Studies demonstrate that diminished PGC-1α levels cause aberrant neuronal dynamics within the primary somatosensory cortex (S1), exacerbating nociceptive behaviors following neural injury (47). This mechanism likely underlies the heightened vulnerability to chronic pain observed in older populations.

The concept of “inflammaging” is key to understanding CPSP in the older adults, but most studies haven’t analyzed cytokine levels by age. To validate the link between perioperative inflammation and CPSP in older patients, large, long-term studies are needed.

### 3.3 Genetic and epigenetic factors in aging

Genetic factors significantly influence individual susceptibility to chronic pain in the older population. Specific gene variants can impact pain sensitivity, inflammatory responses, and the adaptation of the nervous system.

Genes associated with pain perception are crucial in the transmission and perception of pain. Notably, the A118G polymorphism in the OPRM1 gene has been correlated with variations in pain severity and opioid consumption (48). Furthermore, gene variants may contribute to individual differences in pain perception, which are intricately linked to the underlying biological mechanisms of pain (49).

Genes related to inflammation and immunity have been implicated in chronic pain susceptibility. Research indicates that polymorphisms in specific immune-related genes are associated with an increased risk of chronic pain (50). Variations in specific cytokine genes–including those encoding tumor necrosis factor-α (TNF-α), interleukin-6 (IL-6), interleukin-1β (IL-1β), and interleukin-10 (IL-10)–can affect an individual’s pain response and resilience (51). These genetic variations not only impact pain perception but may also influence the duration of chronic pain by modulating inflammatory responses and neural adaptation.

Genome-wide association studies (GWAS) demonstrate significant genetic overlap between chronic pain and major depressive disorder, with neuroticism acting as a key modulator (52). Current evidence suggests that depression comorbid with chronic pain may constitute a distinct genetic subtype of depression. This underscores the necessity of integrating personality traits and stress-related factors when investigating the genetic architecture of complex heterogeneous phenotypes. Genetic predispositions and environmental exposures interact bidirectionally to amplify or mitigate pain symptoms. Research showed that nerve injury induces upregulated expression of epigenetic regulators such as chromodomain Y-like protein (CDYL) in peripheral sensory neurons. CDYL-mediated transcriptional repression of the potassium channel gene Kcnb1 enhances neuronal hyperexcitability and promotes pain sensitization (53). These gene-environment interactions likely contribute significantly to chronic postsurgical pain development in geriatric patients.

Epigenetic regulation offers a promising avenue for understanding chronic pain in aging, but current knowledge is mostly based on preclinical models or non-surgical neuropathic pain studies. Translational research on epigenetic markers in blood or tissue from older surgical patients is necessary for biomarker discovery and understanding mechanisms.

In summary, the development of CPSP in older adults involves complex biological interactions linked to aging. Current understanding often relies on data from younger individuals or preclinical models, highlighting a gap in research specific to older surgical patients. Table 1 outlines the main mechanisms, emphasizing the urgent need for studies focused on the unique aspects of aging related to postsurgical pain.

**TABLE 1:** Key mechanisms of CPSP in older adults: evidence levels and knowledge gaps.

Mechanism/pathway	Age-related changes	Evidence in general CPSP	Evidence in older adults	Key knowledge gaps
Central sensitization	Reduced inhibitory neurotransmission; altered NMDA receptor function	Strong (human/animal studies) (26, 28)	Limited (extrapolated from mixed-age cohorts)	Whether aging accelerates or dampens central sensitization post-surgery
Peripheral sensitization	Slower nerve regeneration; decreased ion channel expression	Strong (human/animal studies) (26, 27)	Moderate (inferred from neuropathic pain studies)	Role of age-related axon degeneration in CPSP persistence
Neuroinflammation (glial activation)	Increased baseline pro-inflammatory cytokines	Strong (animal models) (30, 31)	Limited direct evidence	Temporal dynamics of neuroinflammation in aged surgical populations
Immune dysregulation (Treg/cytokines)	Immunosenescence; altered Treg function	Emerging (animal models) (43, 44)	Scarce	How immunosenescence modulates CPSP risk and recovery
Mitochondrial dysfunction/oxidative stress	Decreased PGC-1α; increased ROS	Emerging (animal models) (46)	Indirect (associated with frailty)	Causal link between mitochondrial health and CPSP in older adults
Epigenetic regulation (e.g., CDYL)	Age-related epigenetic drift	Emerging (animal models) (53)	No direct human studies	Whether age-specific epigenetic signatures predict CPSP
Genetic polymorphisms (e.g., OPRM1)	Polymorphism frequency may vary with age	Moderate (mixed-age GWAS) (48, 52)	Limited age-stratified analyses	Gene-environment interactions in older surgical patients

Data from these studies (26–28, 30, 31, 43, 44, 46, 48, 52, 53). CPSP, chronic postsurgical pain.

### 3.4 The biopsychosocial model in geriatric pain

**FIGURE 1:**
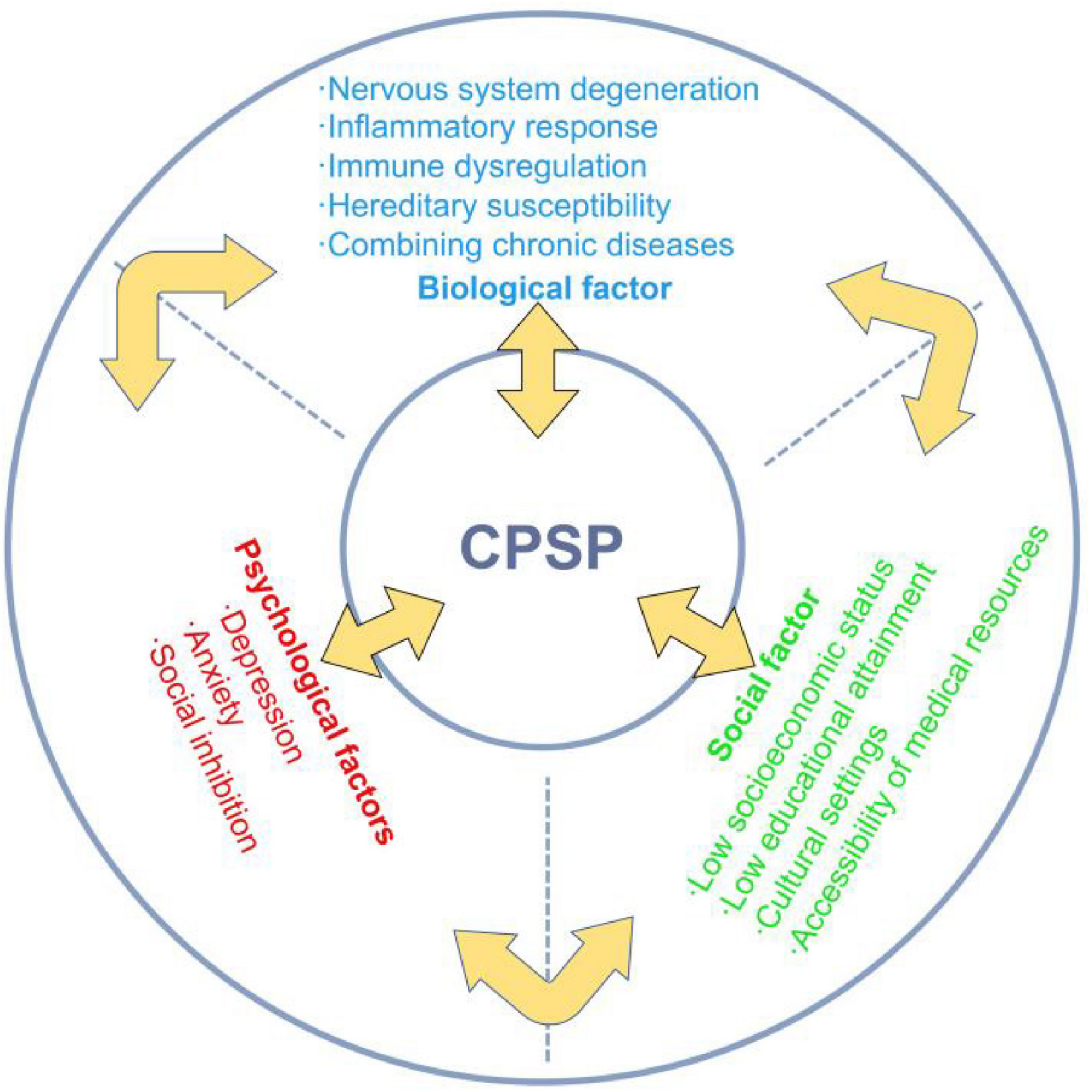
Summarizing the BPS model and its factors.

The Biopsychosocial Model (BPS Model) offers a comprehensive framework for understanding and managing chronic pain in older adults. This model underscores that diseases and symptoms are influenced not only by biological factors but also by the interplay of psychological and social factors (54). Its significance in the study of chronic pain among the older adults lies in its ability to elucidate the complexity of pain and to inform multidimensional treatment approaches (Figure 1).

Psychological factors are crucial in the experience and management of chronic pain among older adults. Current research suggests that individuals exposed to highly stressful, threatening, or ineffective environments may develop increased sensitivity and hyper-reactive responses, including pain (55). Depression and anxiety are often considered emotional responses to the aversive nature of the sensory experience associated with chronic pain. Epidemiological evidence indicates that depression and anxiety may precede and increase the risk of the onset, severity, or persistence of chronic pain (56–58). Research has demonstrated that anxiety and depression are prevalent among the older adults, potentially intensifying their subjective perception of pain and prolonging its duration (59). An anxious state may result in an increased focus on pain, whereas depression may diminish pain tolerance; both conditions can exacerbate the sensation of pain by influencing neurotransmitter activity. These negative psychological states can adversely affect patients’ functional levels and quality of life, thereby impeding their recovery process. Research suggests that psychological risk factors may significantly impact patients’ pain coping strategies, potentially contributing to the development of chronic postoperative pain (60). A retrospective cohort analysis of 14,1466 patients demonstrated that individuals developing CPSP had a higher risk of subsequent depression compared to those without CPSP (adjusted HR = 1.41; 95% CI 1.35–1.48; *p* < 0.0001), confirming CPSP as a significant independent predictor of incident depression (61). Therefore, prioritizing mental health is essential in the management of chronic postoperative pain among older patients.

In addition to the nuanced effects of psychological factors on chronic pain, it is crucial to examine the intricate role of social influences. Global surgical volume estimates indicate 187.2–281.2 million major procedures were performed in 2004 (approximately 1 surgery per 25 people globally) (62). By 2012, this increased to 266.2–359.5 million procedures, reflecting an absolute growth of 33.6% over 8 years (62). CPSP develops in 10%–50% of surgical patients, with 2%–10% experiencing severe pain (62). The continuous increase in population is also a potential reason for the growth of patients suffering from CPSP. Additionally, recent systematic reviews have demonstrated that low educational attainment and socioeconomic status are consistent predictors of the prevalence and severity of chronic pain, as well as associated disabilities and unfavorable surgical outcomes (63, 64). Socioeconomic factors, including poverty, unemployment, and social isolation, are associated with the global severity of chronic pain (65–67). In a cross-sectional survey, a greater number of people living in remote areas and belonging to minority ethnic groups suffered from chronic pain (1.46, CI 95%, 1.30–1.65; *p* < 0.001). Emma et al.’s systematic review of 41 studies (spanning 17 countries, *n* = 2,161,617) demonstrated significantly elevated chronic pain prevalence and severity among immigrant and ethnic minority populations globally, revealing structural health inequities in pain burden distribution (63).

Chronic postsurgical pain in oldrer patients emerges from a complex interplay of biological, psychological, and social determinants. Biologically, age-related neuroimmune degeneration, genetic susceptibilities, inflammatory dysregulation, and comorbid disease burden collectively heighten nociceptive sensitivity and impede pain resolution. Psychologically, pre-existing affective disorders, maladaptive stress responses, and pain catastrophizing behaviors significantly amplify pain perception, impair coping mechanisms, and prolong recovery trajectories. Socially, structural inequities, including socioeconomic deprivation, limited education, cultural marginalization, and inadequate support systems, correlate with increased pain severity, functional disability, and suboptimal treatment outcomes. Critically, these domains exhibit bidirectional interactions: neuroinflammation may exacerbate depressive symptoms, while socioeconomic stressors can potentiate physiological stress responses. This multifactorial framework underscores the necessity of multidimensional risk assessment in older surgical populations. Effective CPSP mitigation requires integrated strategies addressing biomarker profiling, psychological resilience building, and social determinant optimization to enable personalized intervention paradigms.

**FIGURE 2:**
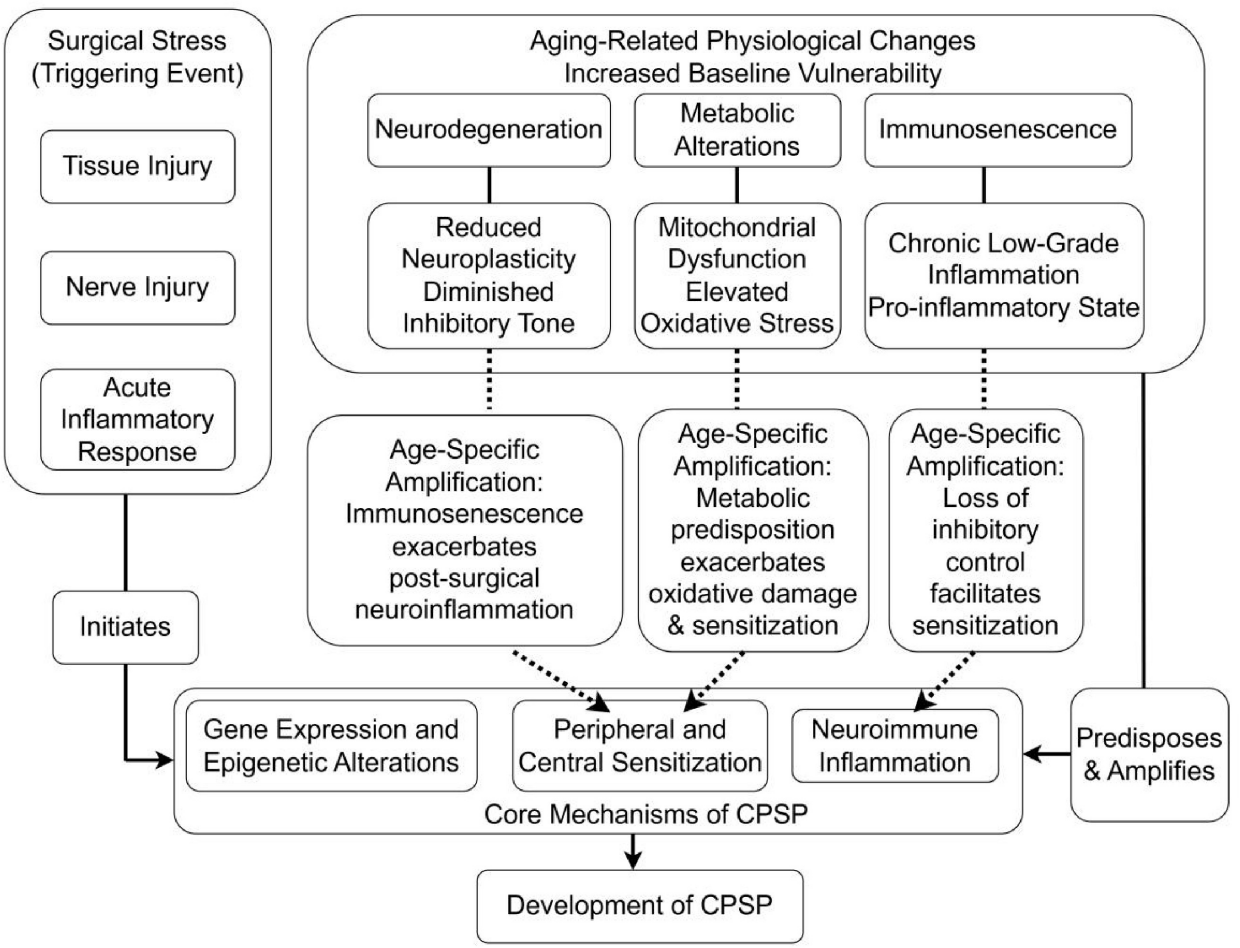
Integrated model of interactions between aging processes and CPSP development.

The complex interactions between age-related physiological decline, surgical stress, and pain chronification can be conceptualized through an integrated pathophysiological framework (Figure 2). This model illustrates how baseline vulnerabilities established by neurodegeneration, immunosenescence, and metabolic alterations interact with the acute stressors of surgery (tissue injury, nerve damage, inflammation) to initiate and amplify the core mechanisms of CPSP (peripheral/central sensitization, neuroimmune inflammation).

These mechanistic insights inform the development of targeted assessment approaches that account for age-related changes in pain expression and perception within the surgical context.

## 4 Surgical context and perioperative considerations

### 4.1 Surgical stress and neuroimmune interactions

Surgical tissue injury initiates a cascade of neuroimmune responses that can lead to persistent pain states. Post-operative inflammation has the potential to result in persistent pain. Clinical studies suggest that plasma concentrations of inflammatory mediators generally change following surgery and may increase immediately thereafter (37–39). Postoperative inflammatory responses can play a critical role in the development of CPSP. This heightened response can adversely affect wound healing and contribute to the induction of chronic pain.

Mitochondrial dysfunction and oxidative stress are increasingly implicated in CPSP pathogenesis. Evidence indicates that mitochondrial impairment drives reactive oxygen species (ROS) accumulation, subsequently inducing neuronal pyroptosis – an inflammatory programmed cell death process that critically contributes to CPSP (46).

### 4.2 Procedure-specific risk profiling

Older patients are subjected to a diverse array of surgical interventions, each presenting distinct risks for the development of postoperative chronic pain. The incidence and severity of CPSP are frequently correlated with the surgical site and the invasiveness of the procedure. Thoracic surgeries, especially those involving the lungs and pleura, are notably associated with a high incidence of chronic pain, which adversely affects the quality of life (68). A prospective cohort study examining open-heart surgery identified preoperative chest pain and elevated immediate postoperative pain scores as significant predictors of chronic pain 6 months following the procedure (69). A retrospective study on chronic pain after donor nephrectomy found that 33% of patients reported experiencing chronic pain (70). These findings suggest that both the nature of the surgical procedure and the patient’s preoperative condition play crucial roles in the development of CPSP. As the prevalence of complex surgical procedures among older adults continues to rise, it becomes imperative to elucidate the relationship between various surgical types and the risk of CPSP in order to develop customized pain management strategies. Comprehensive pre-operative evaluations and meticulous post-operative care are essential to mitigating the incidence of CPSP and enhancing the postoperative quality of life for older patients. Table 2 presents the incidence rates of CPSP following several common surgical procedures (13, 26, 71–73).

**TABLE 2:** The incidence of CPSP in different types of surgery.

Type of surgery	Chronic pain up to 12 months (%)	Incidence of all cpsp (%)
Thoracotomy	41.2	5–71
Sternotomy	27	7–50
Knee arthroplasty	18.4	13–44
Hip arthroplasty	28	27
Inguinal hernia surgery	29.7	5–63
Mastectomy	43–56 (breast cancer surgery)	11–57
Gynecologic surgery	15–40	Not reported
Lower limb amputation	75	30–85
Cesarean section	Not reported	6–55
Cholecystectomy	Not reported	3–56
Craniotomy	Not reported	7–65
Dental surgery	Not reported	5–13
Abdominal surgery (bowel and colorectal)	Not reported	17–21

Data from these studies (13, 26, 71–73). CPSP, chronic postsurgical pain.

Understanding the surgical context and age-related vulnerabilities provides the foundation for developing comprehensive assessment strategies tailored to older adults.

## 5 Clinical assessment and diagnostic approaches

### 5.1 Predictive modeling for CPSP risk stratification

Numerous studies have been undertaken to develop and validate prediction models for CPSP in older patients, with the objective of identifying individuals at high risk and implementing preventive strategies. A retrospective cohort study involving 577 older patients (aged 65 years and older) who underwent thoracic surgery reported a CPSP incidence rate of 26.9%. The analysis identified that variables including age over 75, body mass index, intraoperative blood loss, length of hospital stay, and preoperative neutrophil count are associated with CPSP. The bootstrap stepwise model demonstrated an area under the curve (AUC) of 0.66 (95% CI, 0.61–0.71) in the observational cohort and 0.64 (95% CI, 0.59–0.69) in the validation cohort (74). The calibration curve indicated strong agreement between predicted and observed risks of chronic postsurgical pain in older adults.

Additionally, a separate prospective study has validated a CPSP risk model that is predicated on six clinical predictors. They applied the generalized linear mixed model generated by the development study (75), which integrates variables including the type of surgery, patient age, physical and mental health status, as well as preoperative pain at both the surgical site and other locations. The findings indicate that this model is capable of effectively identifying approximately 70% of patients at risk for CPSP across diverse patient populations (76).

These studies suggest that prediction models for CPSP in older patients assist clinicians in identifying individuals at high risk and in developing personalized pain management strategies, thereby enhancing postoperative quality of life (74, 76). However, several limitations are evident in these studies. Firstly, the generalizability of these models to surgical procedures not encompassed by the inclusion criteria necessitates further validation. Secondly, certain procedures analyzed demonstrated sex specificity; for instance, the study conducted by Montes et al. exclusively included male patients undergoing hernia repair or thoracotomy (76). Additionally, the retrospective nature of the study design precluded the evaluation of psychological status. Future investigations employing more advanced machine learning techniques could potentially better capture the non-linear interactions among clinical, psychosocial, and genetic factors, thereby achieving greater predictive accuracy compared to traditional statistical models.

### 5.2 Comprehensive geriatric pain assessment

Subjective pain assessment primarily depends on patient self-reports and constitutes a critical component of pain evaluation. Commonly employed instruments include the Visual Analog Scale (VAS), the Numeric Rating Scale (NRS), and the Facial Expression Scale (FPS). These tools necessitate a certain level of communicative and cognitive ability from patients. However, older adults may encounter challenges in accurately articulating their pain due to cognitive impairments or language expression difficulties. Consequently, the selection and application of assessment tools must be carefully tailored to accommodate the specific circumstances of each individual. Consequently, observational pain tools (OPTs) have been devised to bridge this gap. Empirical studies suggest that instruments such as the Pain Assessment in Advanced Dementia (PAINAD) and the Pain Assessment Checklist for Seniors with Limited Ability to Communicate (PACSLAC) are efficacious in assessing pain among cognitively impaired older adults (77, 78). The PAINAD and PACSLAC scales differ mainly in their methods and target groups (Table 3). PAINAD is ideal for quick pain identification and management, while PACSLAC offers greater detection accuracy through detailed behavioral observation, making it better for long-term monitoring (79, 80). These tools integrate multiple behavioral indicators, including facial expressions and body movements, to facilitate a more precise evaluation of pain in situations where self-reporting is not possible.

**TABLE 3:** Comparison of pain assessment tools for older adults with cognitive impairment: PAINAD vs. PACSLAC.

Feature	PAINAD	PACSLAC
Primary objective	To provide a rapid and efficient instrument for pain identification and initial management.	To enable a comprehensive and detailed detection of pain behaviors for longitudinal monitoring.
Target population	Primarily designed for individuals with advanced dementia.	Applicable to a broader range of seniors with various communication limitations.
Assessment domains	Evaluates five core behavioral indicators: 1) Breathing 2) Negative vocalization 3) Facial expression 4) Body language 5) Consolability	Assesses a wide spectrum of behaviors across four categories: 1)Facial expressions 2)Activity/body movements 3)Social/personality factors 4)Physiological indicators/eating/sleeping changes
Typical use case	Clinical quick-check during routine rounds or in response to sudden behavioral changes. Ideal for fast-paced environments like acute care settings.	In-depth evaluation in stable, long-term care settings (e.g., nursing homes) for establishing a baseline and tracking pain over time.
Time required	Very brief (approximately 1–2 min to administer).	More time-consuming due to its detailed nature (approximately 5–10 min).
Key strength	Speed and practicality. Its simplicity facilitates high-frequency use and quick decision-making by frontline clinicians.	Comprehensiveness and accuracy. Its detail-oriented design minimizes the risk of missing subtle pain cues, enhancing detection reliability.
Primary limitation	May overlook subtle or complex pain presentations due to its brevity.	Its length can be a barrier to implementation in time-constrained clinical environments.

The selection between PAINAD and PACSLAC is not a matter of superiority but of contextual appropriateness. The PAINAD scale is optimized for rapid clinical utility, whereas the PACSLAC is engineered for detailed observational accuracy, making it better suited for long-term care planning and comprehensive assessment. Data from these studies (77, 78). PAINAD, Pain Assessment in Advanced Dementia; PACSLAC, Pain Assessment Checklist for Seniors with Limited Ability to Communicate.

#### 5.2.1 Special considerations for geriatric pain assessment

Pain assessment in older adults requires tailored approaches accounting for cognitive, sensory, and functional limitations: (1) cognitively intact older people: standard self-report tools (NRS, VAS) remain valid, though may require larger formats and verbal reinforcement. (2) Mild-moderate cognitive impairment: behavioral observation tools (PAINAD, PACSLAC) supplemented with surrogate reporting from caregivers. (3) Advanced dementia: multimodal assessment combining behavioral observation, physiological monitoring, and trial of analgesic interventions.

Challenges in CPSP-specific assessment: (1) distinguishing new surgical pain from pre-existing chronic pain conditions. (2) Accounting for atypical pain presentations (e.g., silent ischemia, painless pathologies). (3) Integrating functional impact measures (Activities of Daily Living, mobility scales). (4) Incorporating frailty assessment (Clinical Frailty Scale) for risk stratification.

Objective physiological indicators offer an alternative method for pain assessment, particularly in patients with non-verbal or cognitive impairments. Research indicates that physiological signals, such as heart rate variability, skin conductance, and facial electromyography, can be effectively employed to evaluate pain levels (81). These indicators have the potential to augment subjective evaluations, thereby providing a more comprehensive understanding of the patient’s pain experience. For instance, a study investigating the application of wearable technology to monitor patients’ physiological responses post-surgery demonstrated the feasibility of incorporating these objective measures into standard pain assessment protocols (81). Aside from that, the researchers examined the correlation between physiological measures and subjective pain scores, discovering that although a relationship exists, the outcomes of the two assessments may not always align. This disparity underscores the significance of employing multimodal pain assessment approaches in the geriatric population (81, 82).

Research has demonstrated that these devices have the potential to outperform traditional subjective pain assessment methods by monitoring functional clinical indicators, thereby providing more objective outcomes. Advances in modular wearable technology, for instance, now permit the tracking of various health parameters, including aerobic capacity, physical activity, stress levels, and sleep quality. These objective metrics can assist in the management of chronic conditions and offer a more comprehensive evaluation of pain and functional status (83). Furthermore, research on postoperative recovery highlights the promise of wearable sensors. A comparative study of patients undergoing uniportal endoscopic versus open lumbar surgery indicated that those utilizing wearable sensors experienced a more rapid recovery of mobility and benefited from continuous physiological assessment. This methodology not only facilitates the creation of individualized rehabilitation protocols but also supports timely interventions throughout the recovery process (84).

In summary, the selection of tools for assessing postoperative chronic pain in older adults should encompass both subjective and objective metrics. Integrating observational pain assessment instruments with physiological indicators enables healthcare professionals to enhance the precision and efficacy of pain management strategies within this demographic.

### 5.3 Diagnostic challenges and differential diagnosis

Diagnosing CPSP in older adults presents a significant challenge due to the multifaceted nature of pain and the concomitant effects of various health conditions. Older patients frequently experience multiple comorbidities, such as osteoarthritis, neuropathic pain, and other chronic pain syndromes. These comorbidities can obscure or exacerbate postoperative pain, complicating both pain assessment and diagnosis. Consequently, differentiating between pain directly attributable to surgical intervention and pain originating from pre-existing conditions becomes increasingly complex.

Cognitive impairments can impede patients’ capacity to effectively communicate their pain levels, resulting in inadequate pain assessment and management. Research indicates that older patients with cognitive impairments are less likely to utilize standardized tools for pain assessment, often leading to delays in receiving appropriate pain interventions (85). Insufficient pain management can exacerbate cognitive decline, elevate the risk of psychosis, and further complicate clinical presentations. Moreover, cognitive dysfunction may affect a patient’s perception of pain and the effectiveness of pain management strategies. Individuals with dementia may demonstrate an atypical response to pain, potentially resulting in misdiagnosis or inappropriate treatment (86).

Chronic postsurgical pain is frequently underdiagnosed or subject to delayed diagnosis in resource-constrained settings and long-term care facilities. The limited availability of diagnostic tools and technologies in these environments constitutes a significant impediment. Advanced diagnostic modalities, such as computed tomography (CT) or magnetic resonance imaging (MRI), are often inaccessible or prohibitively expensive, thereby exacerbating the challenges associated with the precise identification of chronic postsurgical pain (87). Consequently, healthcare providers in these settings are often compelled to depend exclusively on comprehensive history-taking and physical examination for initial assessments, which may elevate the risk of misdiagnosis or overlooked cases.

**FIGURE 3:**
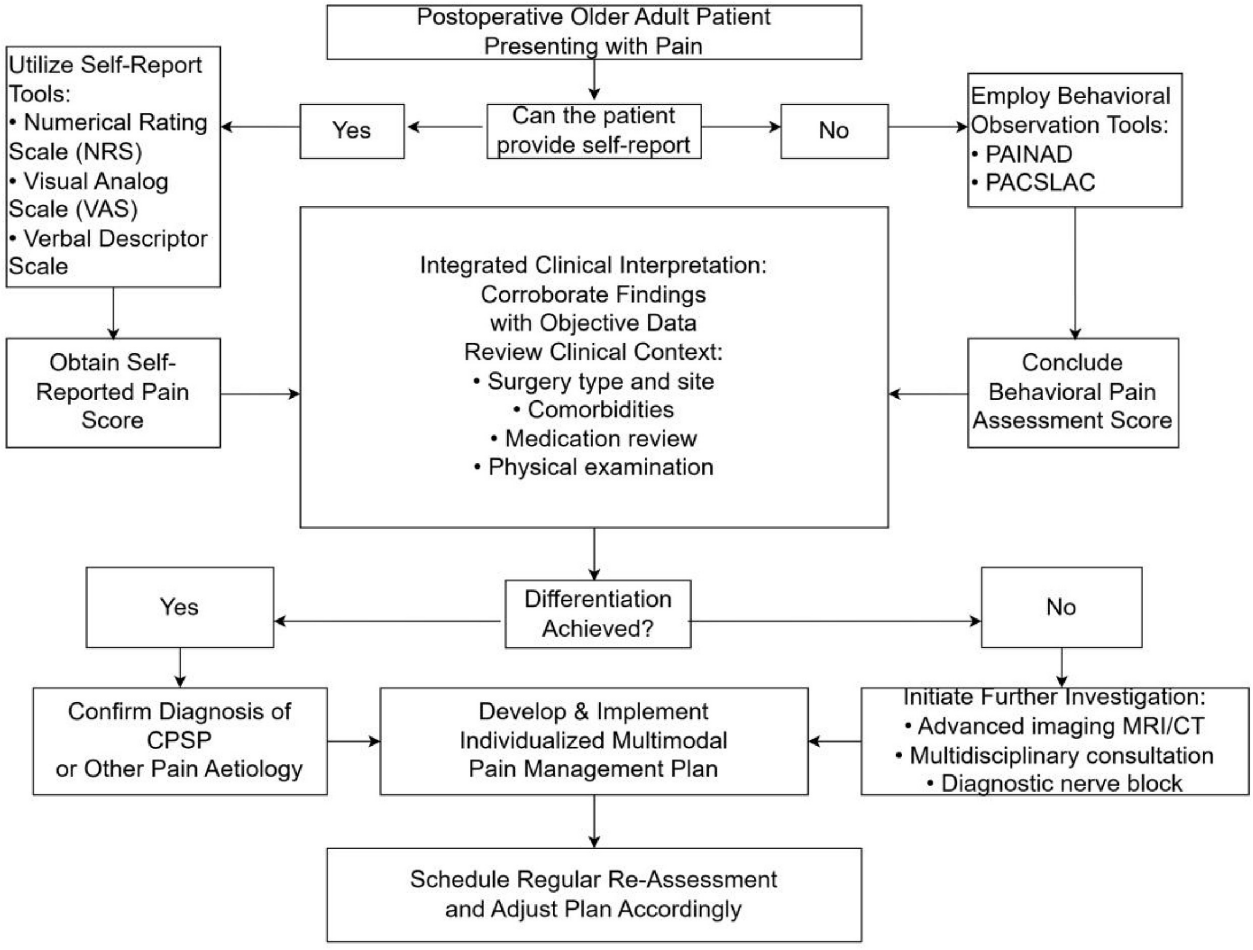
Clinical evaluation flowchart for postsurgical pain in older adults.

The diagnostic complexities associated with postoperative chronic pain in older adults are multifaceted, involving the interplay of comorbidities and cognitive impairments. Overcoming these challenges necessitates a multidisciplinary approach that prioritizes comprehensive evaluation and individualized pain management strategies, thereby enhancing outcomes in the older adult population (Figure 3).

Accurate assessment provides the foundation for implementing personalized management strategies that address the multifactorial nature of CPSP in older adults.

## 6 Management and prevention strategies

### 6.1 Multimodal analgesia and regional techniques

At present, there are no effective pharmacological interventions or preventative measures available to mitigate CPSP in the older adults, owing to the myriad factors influencing its onset. Nonetheless, it is imperative for healthcare professionals to endeavor to minimize the incidence of CPSP and to provide appropriate attention and care to affected individuals. Multimodal analgesia is defined as two or more analgesic modes targeting different receptors along the pain pathway to improve analgesia while reducing side effects (88). Recent research indicates that multimodal analgesia may offer potential benefits in reducing CPSP in patients, with regional block techniques or epidural block techniques serving as the cornerstone of this approach. Research suggests that regional blockade can attenuate nociceptive nerve impulses, modulate glial cell signaling, and diminish neuronal synaptic plasticity (89, 90). Concurrently, local anesthetics possess intrinsic properties that contribute to the reduction of sensitization and exhibit anti-inflammatory effects. A retrospective study demonstrated that patients who underwent general anesthesia exhibited a heightened risk of developing CPSP within 6 months following surgery, in comparison to those who received regional anesthesia (3). Evidence suggests that regional anesthesia is effective in mitigating the incidence of CPSP after procedures such as hysterectomy, cesarean section, and total knee replacement surgeries (91–93). Moreover, a recent observational study suggested that the use of regional blocks may reduce postoperative opioid consumption, especially in patients with higher pain thresholds (94). The administration of elevated intraoperative opioid doses, especially remifentanil, may lead to intensified acute postoperative pain and increased analgesic consumption, potentially attributable to opioid-induced hyperalgesia (95). Employing regional block techniques can mitigate intraoperative opioid requirements and prevent the heightened pain sensitivity associated with high-dose opioid administration, thereby offering a more advantageous strategy for the prevention of CPSP in older patients.

Regional block constitutes a critical component of multimodal analgesia, with pharmacological agents also playing an indispensable role. Numerous drugs can counteract the receptors within pain pathways that are activated by surgical stress. These primarily include local anesthetics, NMDA receptor antagonists, Non-steroidal anti-inflammatory drugs (NSAIDs), and anticonvulsants, which target various channels or receptors and exert effects on both peripheral and central regions (96). Similar to regional blocks, these pharmacological agents aim to inhibit the activation of peripheral neurons, mitigate detrimental stimulation of the central nervous system, and ultimately achieve effective pain control.

### 6.2 Pharmacological management with geriatric considerations

#### 6.2.1 Lidocaine infusion therapy

Drawing upon evidence from multiple randomized controlled trials and retrospective studies, lidocaine, a local anesthetic, emerges as a potentially valuable alternative for the management of chronic neuropathic pain in select patient populations. The continuous infusion of lidocaine during the perioperative period has been shown to extend pain control by attenuating the inflammatory response, diminishing the perception of neuropathic pain, and reducing central sensitization (97). The mechanism of action may extend beyond the inhibition of voltage-gated sodium channels (VGSCs) to encompass effects on hyperpolarization-activated cyclic nucleotide-gated channels, transient receptor potential ion channels, and specific G-protein-coupled receptors (GPCRs) (97, 98). Harriet’s systematic review on lidocaine examined 12 randomized controlled trials with patients averaging over 60 years old, including seven in surgical settings (99). These seven studies reported no adverse effects from lidocaine. However, a weight-based dosing of 2 mg/min for patients under 70 kg and 3 mg/min for those 70 kg or more is discouraged in the older adults due to inconsistent plasma concentrations, potentially affecting analgesic effectiveness and increasing toxicity risk (99). Two distinct lidocaine dosing regimens were utilized across these studies. The first protocol entailed a continuous intravenous infusion at a rate of 1 mg/kg/h, which was initiated at the conclusion of surgery and sustained for a duration of 24 h (100). The second regimen comprised an intravenous bolus dose of 1.5–2 mg/kg, administered either at the induction of anesthesia or 30 min prior to the commencement of surgery. This was followed by a continuous intraoperative infusion at a rate of 1.5–3 mg/kg/h. The infusion was discontinued at various time points, including 30 min before the completion of skin closure (101), at the conclusion of wound closure (102), or 1–2 h post-suturing (103–106). Intravenous administration of lidocaine has demonstrated benefits in breast and abdominal surgeries, with a recent meta-analysis suggesting that its continuous infusion reduces the incidence of chronic pain 3–6 months following surgery (97, 107).

#### 6.2.2 Ketamine and NMDA antagonism

Ketamine is the most extensively studied NMDA receptor antagonist for pain management, as evidenced by numerous systematic reviews and meta-analyses. Research suggests that ketamine may be effective as an adjunctive therapy for both acute and non-cancer chronic pain, although its mechanism of action in chronic postoperative pain remains inadequately understood (108–110). A cochrane database system review demonstrated that the administration of intravenous ketamine at least 24 h following surgery resulted in a reduction of chronic pain at both 3 and 6 months postoperatively (109). This analysis encompassed 14 randomized controlled trials examining the perioperative administration of ketamine. In the majority of studies, ketamine was administered as a pre-operative bolus ranging from 200 to 500 μg/kg, followed by an intraoperative infusion of 50–300 μg/kg/h, or in some cases, no intraoperative dosing was applied. Esketamine, the S-enantiomer of ketamine, demonstrated more potent antidepressant effects and a more favorable side-effect profile in comparison to ketamine (111). Several randomized controlled trials have validated the safety of administering intraoperative esketamine at doses below 0.5 mg/kg/h, in conjunction with postoperative analgesia using 0.72 mg/kg of esketamine (112, 113). A systematic review demonstrated that the administration of intravenous ketamine during perioperative thoracotomy was associated with a reduction in the incidence of acute pain, however, the evidence supporting its efficacy in preventing CPSP remains limited (114). A recent systematic review and meta-analysis provides further evidence supporting the potential of ketamine in both preventing and treating CPSP (115). The authors propose that ketamine may mitigate the risk of CPSP by modulating affective and mood-related disorders associated with pain chronification. Consequently, further research is required to address existing limitations, including the need for larger sample sizes, diverse population studies, and variations in administration timing and routes (108, 116).

#### 6.2.3 Adjuvant medications

Additional pharmacological agents encompass anticonvulsants and NSAIDs. Within the category of anticonvulsants, gabapentin and pregabalin are prominent examples. Earlier studies suggested that gabapentin and pregabalin could potentially reduce the risk of chronic postoperative pain (117). However, recent meta-analyses have found no evidence to support the efficacy of these drugs in preventing chronic postoperative pain when used perioperatively (118, 119). NSAIDs are effective in alleviating acute pain and play a crucial role in multimodal analgesia. However, it is noteworthy that randomized controlled trials have yet to demonstrate a substantial impact of these medications on the mitigation of chronic postoperative pain (116, 120). In the context of older patients, especially those with an elevated risk of bleeding, it is advisable to administer NSAIDs at reduced dosages with prolonged intervals between doses (121). Chang’s review identified two primary findings. Firstly, restricting NSAID use to the postoperative period, without pre- or intraoperative administration, and maintaining it at the lowest effective dose for a brief duration, did not elevate the risk of surgical bleeding complications (122). Secondly, the short-term use of NSAIDs for postoperative analgesia did not seem to increase the risk of acute kidney injury in patients with normal baseline renal function (122). Moreover, the current literature does not provide conclusive evidence regarding the clinically significant adverse effects of short-term NSAID administration in patients with pre-existing renal impairment (122).

### 6.3 Integrative and non-pharmacological approaches

Acupuncture, a traditional Chinese therapeutic practice, has garnered increasing attention in recent years for its efficacy in the prevention and management of postoperative pain. Acupuncture modulates pain through several neurochemical mechanisms. It activates opioid peptide neurons in the brain and spinal cord, enhances the expression of endogenous opioid peptides, and upregulates opioid receptors within cerebral nuclei. Additionally, acupuncture promotes central 5-hydroxytryptamine (5-HT) synthesis while inhibiting peripheral 5-HT release, resulting in suppressed pain transmission and reduced pain sensitization (123, 124). Furthermore, acupuncture aids in regulating pain conduction by helping to maintain the balance between excitatory neurotransmitters, such as glutamate and aspartate, along with their receptors, and inhibitory neurotransmitters, including γ-aminobutyric acid (GABA) and glycine, and their corresponding receptors (125). A randomized controlled trial investigating acupuncture for neuropathic pain following breast cancer surgery reported significant clinical improvements. Patients in the acupuncture group received 18 sessions over 8 weeks. Compared with the control group, they demonstrated a significantly greater reduction in Brief Pain Inventory-Short Form (BPI-SF) scores (−1.1 ± 1.7 vs. 0.3 ± 1.5; *p* = 0.03) (126). Empirical research suggests that acupuncture substantially mitigates postoperative pain. A systematic review and meta-analysis found that acupuncture may be more effective than pharmacological treatments in reducing pain related to lumbar disk herniation, and was associated with fewer adverse events (127). A study examining the efficacy of acupuncture for chronic pain within a military cohort demonstrated significant improvements in pain scores among patients utilizing electrostimulation acupuncture (128). Acupuncture not only mitigates pain but also potentially exerts a beneficial influence on psychological disorders associated with chronic pain. Research suggests a bidirectional relationship between chronic pain and psychological disorders. As a psychosomatic intervention, acupuncture may effectively alleviate psychological issues resulting from chronic pain (129). In the context of postoperative pain management, acupuncture has shown promise in aiding patients to more effectively manage pain and facilitate recovery. Although acupuncture has demonstrated potential in the management of postoperative pain, there is a paucity of research regarding its efficacy in CPSP. This underscores the need for additional high-quality randomized controlled trials to substantiate its effectiveness and safety.

### 6.4 Multidisciplinary geriatric pain management

Effective CPSP management in older adults requires a multidisciplinary approach that addresses the unique physiological, psychological, and social needs of this population. This includes: (1) geriatrician-led comorbidity optimization. (2) Pain specialist-guided medication management. (3) Physical therapist-supervised functional restoration. (4) Occupational therapist-directed activity modification. (5) Social worker-supported resource navigation. (6) Pharmacist-conducted medication reconciliation.

Special Considerations for Frail Older Adults: (1) polypharmacy management through systematic deprescribing. (2) Functional status preservation through early mobilization. (3) Cognitive and mood disorder screening and management. (4) Social support system assessment and caregiver education.

Table 4 illustrates the perioperative risk factors, assessment, prevention, and treatment strategies for chronic postsurgical pain in the older population (16, 130).

**TABLE 4:** Perioperative risk factors, assessment, prevention and treatment of CPSP in older adults.

Timing	Risk factor	Assessment	Prevention and treatment
Pre-operative	Comorbidities; Psychosocial factors: decreased immune function, anxiety, depression, gene mutation, etc. Type of surgery	Pain questionnaire survey; Psychological and functional assessment; Prediction model	Pain management education; Preemptive analgesia
Intra-operative	Surgical stimulation intensity; Nerve injury; High doses of opioids	Surgical quality assessment; Intraoperative medication evaluation	Personalized multimodal analgesia; Regional or intraspinal block; Reducing opioid use
Post-operative	Comorbidities: cognitive impairment, etc. Peripheral and central sensitization; Psychosocial factors: inflammatory response, economic burden, living alone, education level, depression, anxiety, etc.	Subjective scale evaluation and objective physiological index comprehensive evaluation; Multidisciplinary collaborative evaluation	Postoperative nursing guidance; Pain anticipation management; Multidisciplinary cooperation; Developing personalized pain control strategies

Data from these studies (16, 130). CPSP, chronic postsurgical pain.

## 7 Conclusion

Chronic postsurgical pain in older adults is a multifactorial condition influenced by biological, psychological, and social determinants. This review synthesizes evidence underscoring the necessity of an integrated, interdisciplinary approach to effectively address CPSP in the aging population. Key components of such an approach include collaboration among geriatricians, pain specialists, anesthesiologists, psychologists, physical therapists, and social workers to deliver personalized, multimodal care.

Moving forward, several actionable strategies are recommended. First, policy initiatives should advocate for the integration of CPSP management into standard geriatric care protocols, ensuring that pain assessment and prevention are routine in perioperative care. Second, the implementation of structured, long-term follow-up systems utilizing telehealth and wearable technology for remote pain monitoring can enhance postoperative surveillance and facilitate timely interventions. Additionally, clinical practice should prioritize the use of validated assessment tools tailored to older adults, especially those with cognitive impairments, and embrace multimodal analgesic regimens that minimize opioid exposure.

Technological innovations, including electronic health record integrations and mobile health applications, offer promising avenues for improving pain tracking and patient engagement. Further research should focus on elucidating the molecular mechanisms of CPSP, validating predictive models incorporating genetic and psychosocial variables, and conducting large-scale randomized trials to evaluate targeted interventions.

This review highlights the critical need to reconceptualize CPSP management through a geriatric-focused lens, yet it is limited by the heterogeneity of existing studies and a reliance on observational data. Despite these constraints, this work contributes to the growing discourse on geriatric pain management by providing a comprehensive framework that bridges clinical evidence with practical strategies aimed at improving outcomes in older surgical patients.
